# Nitric Acid in Injuries to the Spine

**Published:** 1889-08

**Authors:** B. Simmons

**Affiliations:** Sydney, N. S. W.


SYDNEY, N. S. W. B. SIMMONS, M. D.

---

### NITRIC ACID IN INJURIES TO THE SPINE.

A severe injury resulting in mischief to the spine is often followed by most troublesome and varied disturbances of the system, and each case must, of course, be treated in strict accordance with the symptoms present. Arn., Rhus, Calc., Hyper., and other medicines are frequently required, but I wish to call attention to Nit-ac., which has in my experience been frequently indicated, and it has helped some cases more than any other agent. After a severe shock to the spine, *a profuse perspiration on the hands and feet often breaks out.* When this symptom is present Nit-ac. should be studied, as it will probably prove to be the simillimum.

